# Does intestinal epithelial integrity status in response to high-protein dairy milk beverage with or without progressive resistance training impact systemic inflammatory responses in an active aging population?

**DOI:** 10.1371/journal.pone.0274210

**Published:** 2022-09-02

**Authors:** Zoya Huschtscha, Pascale Young, Alexandra Parr, Judi Porter, Ricardo Costa

**Affiliations:** 1 Department of Nutrition Dietetics & Food, Monash University, Notting Hill, Victoria, Australia; 2 School of Exercise and Nutrition Sciences, Deakin University, Melbourne Burwood Campus, Burwood, Victoria, Australia; The University of British Columbia, CANADA

## Abstract

Increased resting systemic anti-inflammatory responses have previously been reported after a period of progressive resistance training (PRT) with daily consumption of a high-protein dairy beverage. The study aimed to investigate the independent and combined effects of consuming a high protein dairy milk beverage with or without a PRT on markers of intestinal epithelial integrity and selected systemic inflammatory responses in active older (≥50 yrs) adults. Thirty two (males n = 24, females n = 8) active older adults [mean (SD): Age 62 (7) years, weight 74.2 (14.0) kg, height 1.73 (10.0) cm, BMI 24.9 (4.0) kg/m^2^, and body fat mass: 25.8 (9.1)%)], that reported exercising ≥3/week (211 (91) min/week) were randomly allocated into one of four groups: dairy milk (DM), exercise and dairy milk (EX+DM), exercise alone (EX), and control (CON). Groups with EX underwent 12-weeks whole-body PRT program (x3 sessions/week), groups with DM consumed the beverage twice daily (30g protein/day), and CON was required to carry out their *ad libitum* dietary and exercise habits. Plasma concentrations of CRP, IL-1ß, IL-1ra, LBP, and sCD14 were determined by ELISA from samples collected at weeks 0, 6, and 12. Data were analyzed (SPSS v25.0) for group and time differences using a two-way repeated-measures ANOVA with post hoc analysis. No significant differences were observed for any of the measured plasma biomarkers. The previously observed increase in anti-inflammatory cytokine response is likely due to a muscular cellular response and not an indication of intestinal epithelial integrity disturbance and/or subsequent translocation of luminal originated pathogenic bacterial compounds.

## Introduction

The age-related decline in skeletal muscle mass, skeletal muscle strength, and physical performance is known as sarcopenia [1, 2]. This gradual and generalized loss commences from as early as the 4^th^ or 5^th^ decade of life, and is considered one of the most critical factors implicating the progression of loco-motor functional decline, frailty, and disability with aging [3]. Despite the majority of the current literature focusing on frail and institutionalized older adults, endurance based active older adults still show signs of declining physical function with increasing age despite often exceeding the physical activity guidelines and exercising significantly more than sedentary older adults (>150 min/week of moderate intensity or 75 min/week of vigorous intensity- or combination) [4, 5].

Chronic low-grade inflammation, a term known as ’*inflammaging’* is indicated by a chronic state of modestly disturbed plasma levels of systemic inflammatory mediators. These include increases in tumour necrosis factor-α (TNF-α) and interleukin (IL)-6 that are commonly measured in published literature, and to a lesser extent IL-1β; and a reduction of anti-inflammatory cytokines such as IL-10, and to a lesser extent IL-1ra [6, 7]. Elevated inflammatory mediating cytokines (e.g., TNF-α and IL-6) have been associated with lower skeletal muscle mass and reduced strength in healthy community-dwelling older adults (>70 yrs) [8, 9]. Although inflammaging can accelerate skeletal muscle breakdown, undergoing regular physical activity, in particular resistance training has shown to attenuate some of these losses. For example, a significant increase (≥0.5 kg) in skeletal muscle mass was observed in active older adults after a 12-week progressive resistance-training program with or without protein supplementation [5]. This was concomitant with increased anti-inflammatory cytokine responses. However, key pro- and anti-inflammatory mediators, IL-1β and IL-1ra respectively, were not assessed. In accordance with these outcomes, it is widely acknowledged that engaging in regular moderate physical activity can lead to reductions in systematic inflammation as a result of increased anti-inflammatory cytokine responses (e.g., IL-10) [10–14].

Although exercise has shown to promote an anti-inflammatory affect, one aspect that has been overlooked in research models is the impact of intestinal epithelial integrity and the translocation of luminal originated pathogenic bacterial endotoxins into circulation, and their systemic inflammatory effect in active older adults. Firstly, the contribution on luminal originated pathogenic agents into systemic circulation towards low-grade systemic inflammation has been well discussed, albeit in clinical populations (e.g., cardio metabolic risk) [15–17]. Secondly, acute and chronic (i.e., repetitive consecutive days) exercise protocols have shown to increase bacterial endotoxin translocation (e.g., gram-negative endotoxin by LAL endpoint analysis), and increase plasma concentrations of lipopolysaccharide binding protein (LBP) and bacteria detecting soluble CD14 (sCD14) that are indirect biomarkers for detecting bacterial endotoxin lumen to circulation translocation in response to compromise intestinal epithelial integrity, and subsequent systemic inflammatory response, in active populations, dependant on exercise stress load [18–25]. Such exercise-associated intestinal perturbation stem from the circulatory-gastrointestinal pathway of ‘*exercise-induced gastrointestinal syndrome*’ and has the potential to influence the magnitude of systemic cytokine status. It is currently unknown to which degree the integrity of the intestinal epithelium plays a role in the systemic inflammatory response to exercise or nutrition interventions with a focus on resistance training aimed at sarcopenia management in the active aging population.

In light of this, the current study aimed to investigate the changes in markers indicative of intestinal epithelial integrity (e.g., sCD14 and LBP), and their association with systemic inflammatory profile, during a 12-intervention of progressive resistance training, with or without a high protein dairy milk beverage, in active older (≥50 yrs) adults. Considering exercise has the potential to increase bacterial translocation in both acute and chronic exercise protocols, it was hypothesised that following the 12-week progressive resistance training protocol, with or without diary milk beverage intervention, resting circulatory LBP and sCD14 concentrations would increase and result in an increased systemic inflammatory profile compared to the non-exercising groups.

## Materials and methods

The study protocol obtained approval from the Monash University Human Research Ethics Committee (Project number 12812), in accordance with the 2008 Helsinki Declaration for Human Research Ethics. Informed written consent was obtained from all participants before they were enrolled in the trial. The study was registered with the Australian and New Zealand Clinical Trial Registry as ANZCT12618001088235. Data on participants nutritional and hydration regime and status, body composition, skeletal muscle strength and power, functional performance, and hormonal variables from this clinical trial have been previously published elsewhere [5, 27].

### Participants and study design

The results from this study stem from a larger clinical trial that examined the effects of a high protein dairy milk beverage (provided and breakfast and lunch), or without progressive resistance training (PRT) on outcomes of fat-free mass, skeletal muscle strength, and power, and physical performance in a cohort of healthy older adults [5]. The screening process and study design have been previously described [5]. Older adult males and females, ≥50 years, with no age upper-limit, performing exercise training for recreational fitness and/or sports competitions (e.g., endurance runners, aerobic gym goes), ≥3 structured exercise sessions/week, totaling ≥90 min/week of structured exercise duration, plus additional unstructured physical activity that accounted for meeting the Australian physical activity guidelines [26], were recruited from metropolitan Melbourne and surrounding areas in Victoria, Australia. A total of 32 (males *n* = 24, females *n* = 8) active older adults (mean (SD): Age: 62 (7) years, weight: 74.2 (13.9) kg, height: 173 (10) cm, BMI: 24.9 (3.9) kg/m^2^, and body fat mass: 25.8 (9.1)%)) that exercised a reported ≥3/week (211(91) min/week) were randomly allocated into one of four groups: high protein dairy milk beverage alone (DM), exercise and high protein dairy milk beverage (EX+DM), exercise alone (EX), and control (CON). Participants in the DM group were asked to consume 250 ml of the high protein dairy milk at breakfast and lunch time. This provided an additional 15 g of protein per serve (30 g in total per day). Participants in the EX-group attended supervised whole-body progressive resistance training sessions x3 session/week. N = 4 participants were removed from the original data previously published [5] due to incomplete samples from missed blood collection at certain time points. Randomization was carried out by a researcher blinded to allocation and was not involved in the data collection, using a block randomization table scheme (i.e., 4 trials by 16 participants) with stratification by age and biological sex (www.randomizer.org). If in close contact with other participants (e.g., cross over times during exercise testing), participants were asked to not discuss the study participation with anyone involved in the study.

### Intervention

Prior to commencing any physical activity, participants filled out a physical activity readiness questionnaire (PAR-Q), which they self-reported their level of activity including exercise volume and type. Eligible participants were required to attend the laboratory between 07.00am and 09.00am on three separate occasions: baseline (week 0), week 6, and week 12, over the 12-week intervention. All participants were required to avoid strenuous exercise for a 24 h period prior to all laboratory assessments. Height was assessed using a fixed stadiometer (Holtain, Crosswell, Crymych, UK). Body mass (BM) was measured (Seca 515 MBCA, Seca Group, Hamburg, Germany) to the nearest 0.1 kg, using standardized anthropometrical procedures. The EX group underwent a 12-week whole-body PRT program (x3 sessions/week) and the DM beverage was consumed twice daily (30 g protein/day). The control group received no intervention and were asked to carry out their usual exercise and dietary habits. More details of the study control and interventions are outlined in Huschtscha et al. [5].

### Blood collection and analysis

At baseline (week 0), week 6, and week 12, participants were asked to attend the laboratory for sample collection and key variable assessment. Participants arrived at the laboratory between 7:00am and 9:00am in a fasted state and euhydrated [5]. All participants were required to avoid strenuous exercise for a 24-h period prior to all laboratory assessments. Prior to the collection of any physical exertion related variables, a whole blood sample was collected by venipuncture from an antecubital vein into one lithium heparin (6 ml, 1.5 IU/ml heparin) and one K3EDTA (4 mL, 1.6 mg/ml EDTA) vacutainer tube (Becton Dickinson, Oxford, UK). Blood hemoglobin, was determined by the HemoCue system (Hb201, HemoCue AB, Ängelholm, Sweden) from heparin whole blood samples. Hematocrit was determined using the capillary method from heparin whole blood samples and using a microhematocrit reader (ThermoFisher Scientific). Hemoglobin and haematocrit values were used to estimate changes in plasma volume relative to baseline and used to correct plasma variables. The remaining heparin and EDTA whole blood samples were centrifuged at 4,000 rpm (1,500 × g) for 10 min within 15 min of sample collection. Aliquots of plasma were placed in 1.5-ml microstorage tubes and frozen at -80°C until analysis, except 2 × 50μl plasma heparin samples which were used to determine plasma osmolality [5].

Plasma concentrations of C-reactive protein (CRP) (HK369, Hycult Biotech, Uden, Netherlands), IL-1ß (ELH-IL1b, Raybiotech Life, Peachtree Corners, GA, USA), IL-1ra (ELH-IL1ra, Raybiotech), LBP (HK315, Hycult Biotech, Uden, Netherlands), and sCD14 (HK320, Hycult Biotech) were determined by ELISA, as per manufacturer’s instructions. The CV for CRP and systemic inflammatory cytokine profile was ≤16.8% and ≤12.6%, respectively; and for intestinal epithelial integrity markers was ≤4.9%. A subset of systemic inflammatory cytokine responses (i.e., TNF-α, IL-2, IL-6, IL-8, and IL-10 has previously been published elsewhere [5].

### Statistical analysis

Confirmation of adequate statistical power *a priori* for the primary research are previously described [5]. Participants and researchers at the time of sample and data collection were unaware that further analysis would be undertaken to investigate the experimental design on markers of intestinal epithelial integrity and additional inflammatory cytokine responses. However, based on the statistical test, mean, standard deviation, and effect size of epithelial integrity and systemic inflammatory cytokine values from primary and supportive research [5, 20–25], and applying a standard alpha (0.05) and beta value (0.80), the current participant sample size and group distribution is estimated to provide adequate statistical power (power* 0.80–0.99) for detecting significant primary variable differences within and between groups (G*Power 3.1, Kiel, Germany). Only participants with full data sets were used in the data analysis. Data were checked for distribution using Shapiro-Wilks test of normality. Variables were examined using a two-way repeated-measures ANOVA with a matrix including group (DM, EX+DM, EX, and CON), time (baseline (week 0), week 6, and week 12). Assumptions of homogeneity and sphericity were checked, and when appropriate, adjustments to the degrees of freedom were made using the Greenhouse-Geisser correction method. Significant main effects from the repeated measures ANOVA were further analyzed using a post hoc Tukey’s HSD test to determine between-subject group differences. Correlation analysis were examined between each marker at each time points for absolute change and for % change at different time points, using a Pearson’s or non-parametric Spearman’s correlation analysis, where appropriate. Additionally, Cohen’s *d* ((M_1_ –M_2_) / s_pooled_) was applied to determine the magnitude of effect size for significance differences, with *d ≥* 0.2 for small, *d ≥* 0.5 for medium and *d ≥* 0.8 for large effect size. Statistics were analyzed using SPSS statistical software (V.25.0, Chicago, Illinois, USA) with significance accepted at *P*≤ 0.05.

## Results

There were no significant differences in the baseline characteristics variables between groups (Table 1).

**Table 1:** Participant characteristics at baseline (week 0).

	DM	EX	EX+DM	CON	p-value
	n = 7	n = 8	n = 8	n = 9	
n = males	6	7	4	7	
n = females	1	1	4	2	
Age, years	60.0 (54.2 to 64.0)	59.1 (51.3 to 67.0)	61.4 (55.0 to 68.1)	55.4 (51.4 to 61.3)	0.372
Height, m	1.80 (1.65 to 1.90)	1.76 (1.68 to 1.84)	1.70 (1.61 to 1.73)	1.70 (1.62 to 1.80)	0.123
Body mass, kg	77.0 (62.2 to 91.4)	77.0 (60.5 to 93.4)	70.2 (61.2 to 79.3)	71.1 (65.2 to 77.0)	0.669
BMI, kg/m^2^	24.1 (22.0 to 26.5)	25.0 (21.5 to 28.3)	24.3 (21.4 to 27.4)	24.4 (23.4 to 25.5)	0.959
Body fat, %	24.6 (19.8 to 29.4)	25.0 (19.0 to 30.4)	30.5 (19.3 to 42.0)	26.0 (22.5 to 29.1)	0.440
Self-reported structured exercise, mins/week	202 (108 to 295)	276 (230 to 321)	185 (100 to 270)	229 (189 to 258)	0.258

Values shown are the mean (95% CI).

DM: dairy milk, EX: exercise, EX+DM: exercise + dairy milk, CON: control.

### Intestinal integrity markers

**Fig 1:**
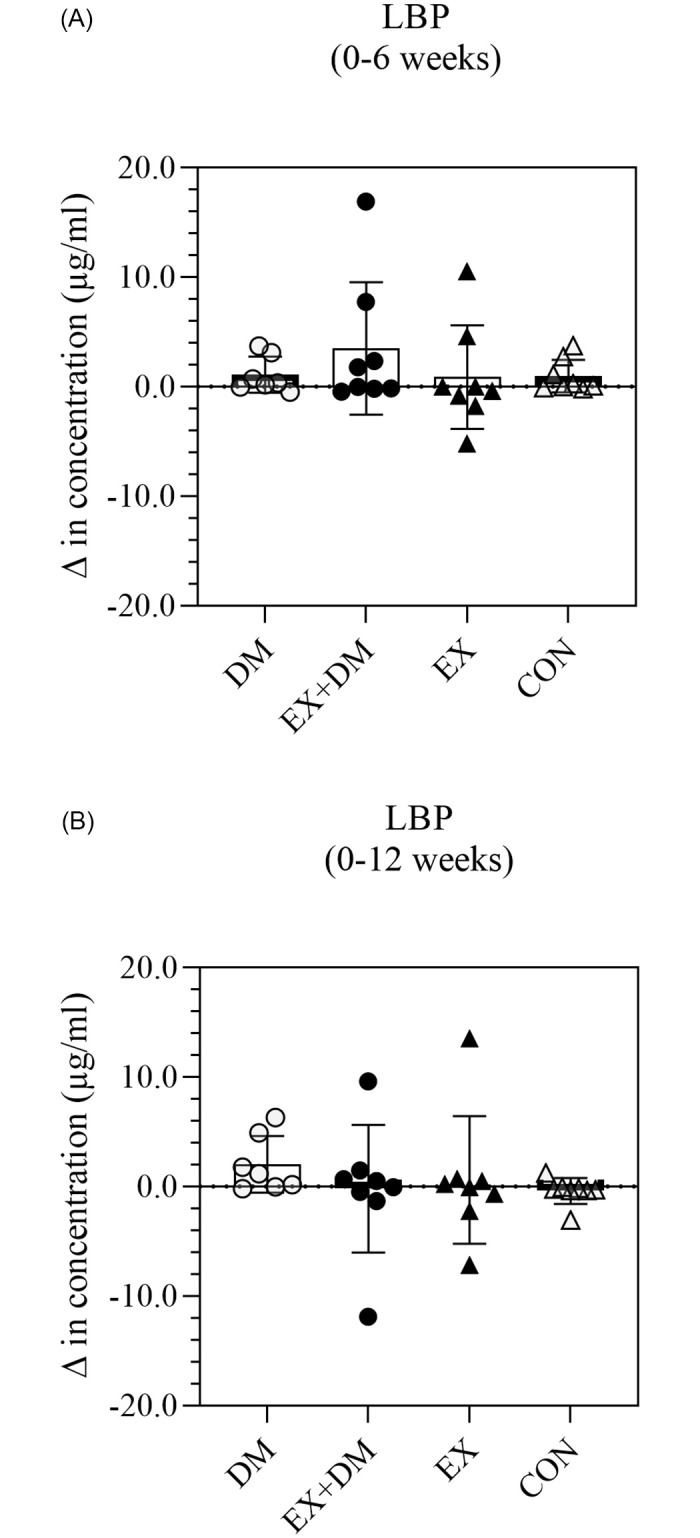
The impact of 12-weeks progressive resistance training, with or without dairy beverage intervention, on changes to resting plasma LPB concentration: (A) 0–6 weeks and (B) 0–12 weeks. Mean ± SEM (*n* = 32): DM ○, EX+DM ●, EX ▲, and CON Δ.

**Fig 2:**
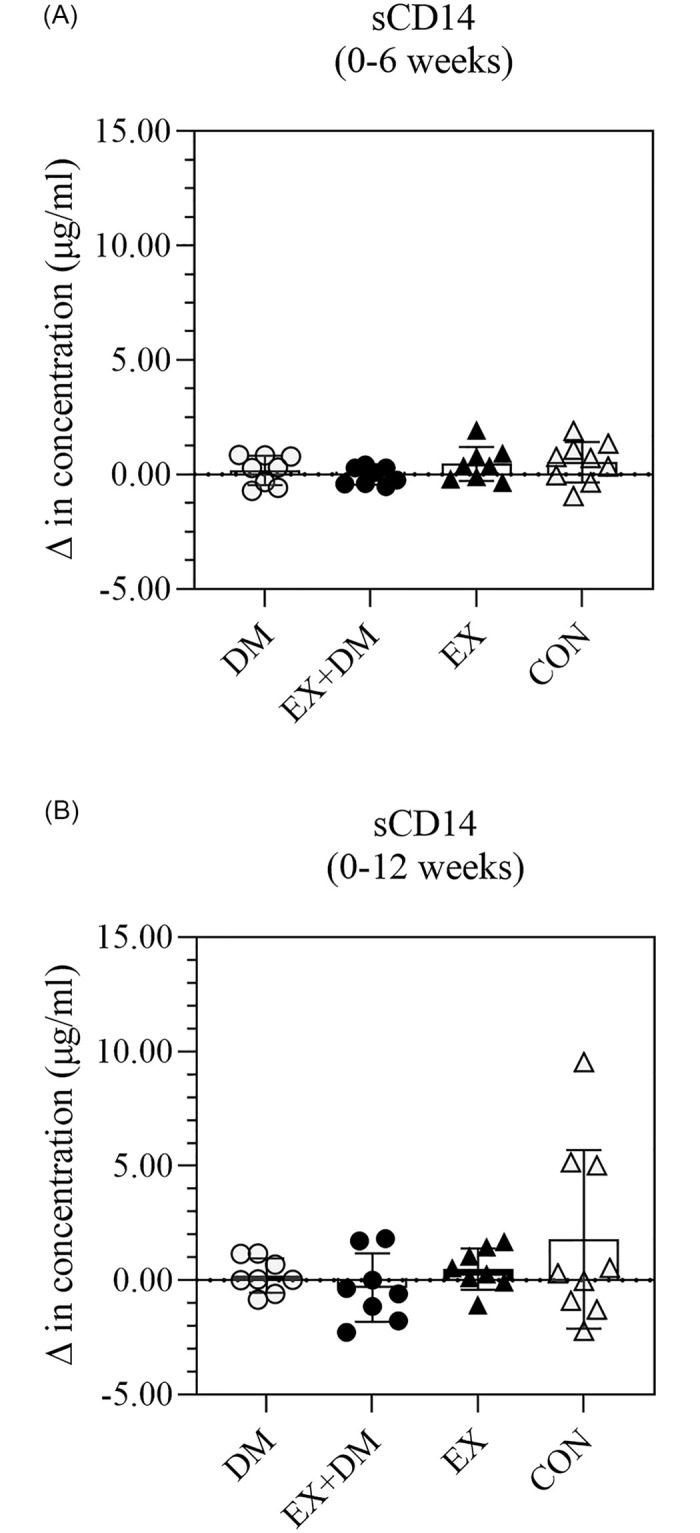
The impact of 12-weeks progressive resistance training, with or without dairy beverage intervention, on changes to resting plasma sCD14 concentration: (A) 0–6 weeks and (B) 0–12 weeks. Mean ± SEM (*n* = 32): DM ○, EX+DM ●, EX ▲, and CON Δ.

There were no significant differences in resting plasma concentrations of LBP and sCD14 observed within and between groups along the intervention period (Figs 1 and 2), with large individual variability within and between groups, and no large effect sizes observed (S1 Table).

#### Systemic inflammatory variables

**Fig 3:**
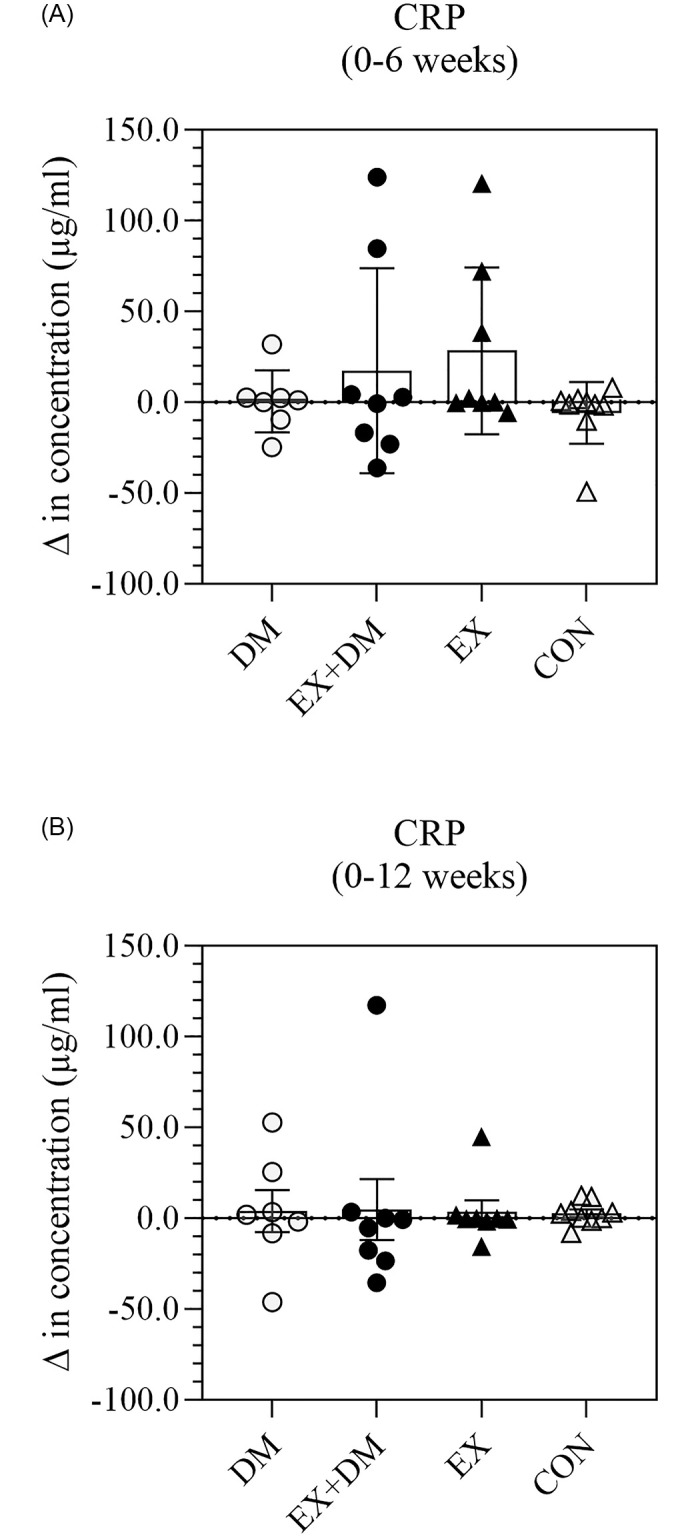
The impact of 12-weeks progressive resistance training, with or without dairy beverage intervention, on changes to resting plasma CRP concentration: (A) 0–6 weeks and (B) 0–12 weeks. Mean ± SEM (*n* = 32): DM ○, EX+DM ●, EX ▲, and CON Δ.

**Fig 4:**
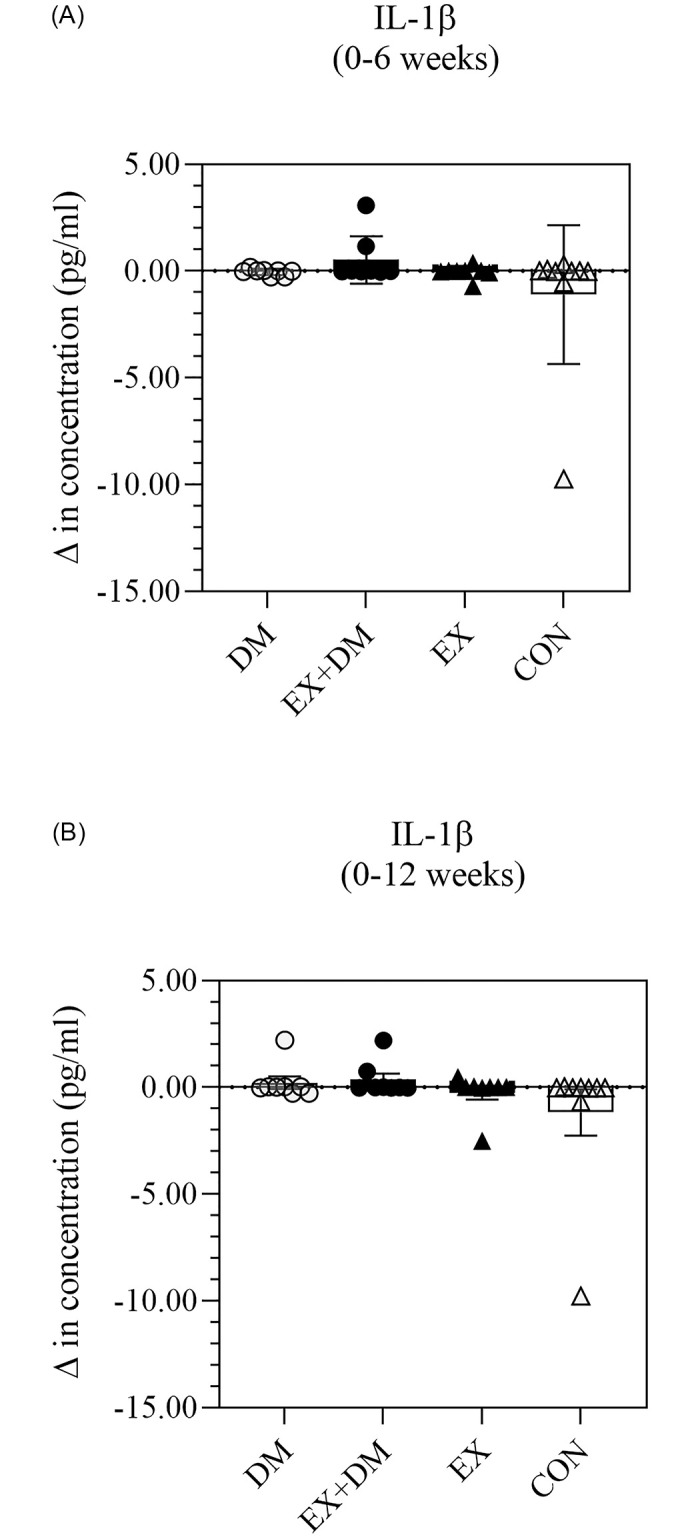
The impact of 12-weeks progressive resistance training, with or without dairy beverage intervention, on changes to resting plasma IL-1β concentration: (A) 0–6 weeks and (B) 0–12 weeks. Mean ± SEM (*n* = 32): DM ○, EX+DM ●, EX ▲, and CON Δ.

**Fig 5:**
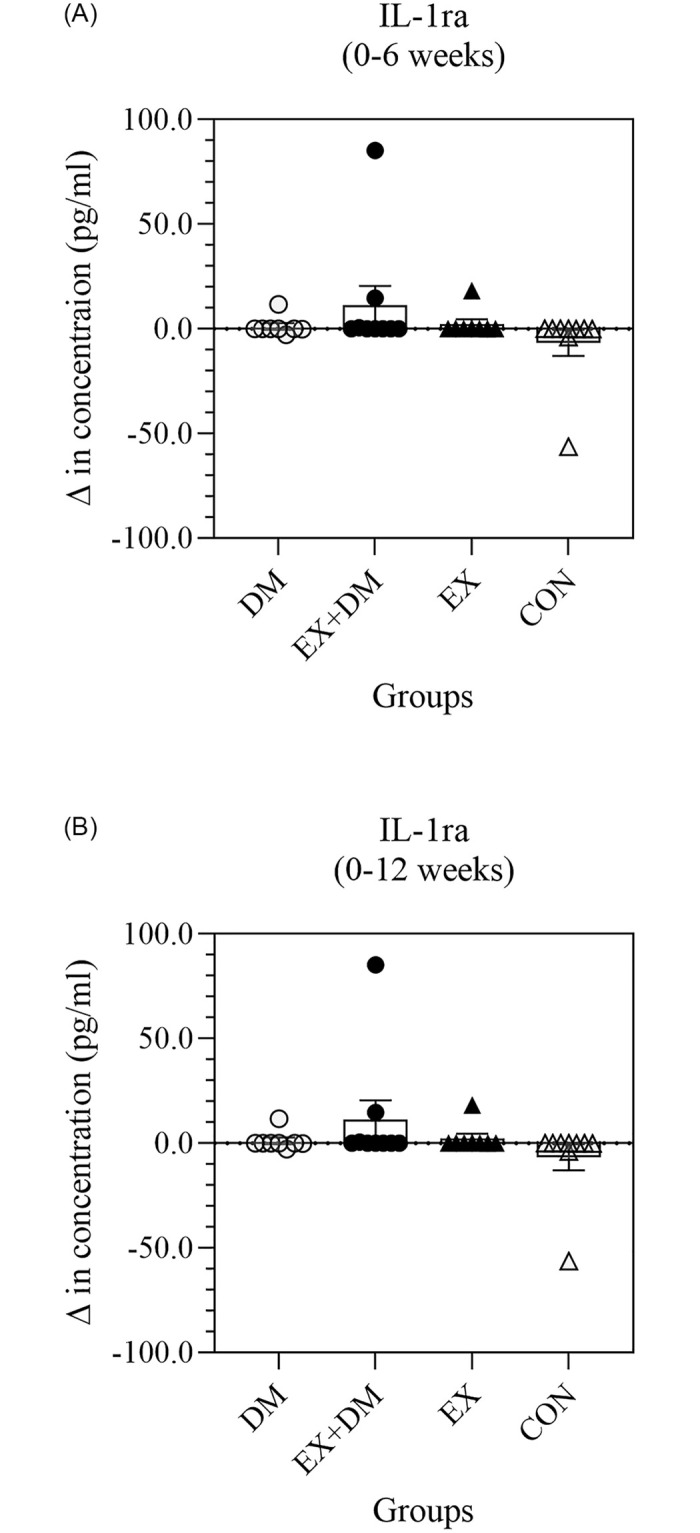
The impact of 12-weeks progressive resistance training, with or without dairy beverage intervention, on changes to resting plasma IL-1ra concentration: (A) 0–6 weeks and (B) 0–12 weeks. Mean ± SEM (*n* = 32): DM ○, EX+DM ●, EX ▲, and CON Δ.

A subset of systemic inflammatory cytokine responses (i.e., TNF-α, IL-2, IL-6, IL-8, and IL-10 has previously been published elsewhere [5]. Additional analysis showed that there were no significant differences in resting plasma concentrations for CRP, IL-1β, and IL-1ra within and between groups (Figs 3–5), with large individual variability within and between groups observed (S1 Table). Only one large effect size was observed for CRP on CON 0 to 12 weeks comparison.

### Correlation analysis

There were no significant correlations between plasma concentrations of LBP and sCD14 with any inflammatory cytokine markers at any time point for the absolute or relative change values, except for a significant negative correlation between absolute plasma IL-1ra with sCD14 at 6 weeks, and a significant positive correlation between relative plasma IL-1β with sCD14 for Δ 6- to 12-weeks (S2 and S3 Tables).

## Discussion

The current study is additional analysis from previously published research [5, 27], and is the first study to explore the impact of intestinal epithelial integrity and the translocation of luminal originated pathogenic compounds into circulation, and their systemic inflammatory effect in active older adults. The current study aimed to investigate the changes in markers indicative of intestinal epithelial integrity (e.g., sCD14 and LBP), and their association with systemic inflammatory profile, during 12-weeks intervention of PRT, with or without a high protein dairy milk beverage, in active older (≥50 yrs) adults. It has previously been reported that PRT and high protein dairy milk beverage increases systemic anti-inflammatory cytokine concentrations (i.e., IL-10) [5]. Contrary to our hypothesis, 12-weeks of PRT, with or without a high protein dairy milk beverage, did not increase circulating sCD14 and LBP concentrations. Moreover, it did not result in an increased additional systemic inflammatory cytokine analysis compared to the non-exercising groups. The main findings from the previously published study [5] showed a significant group*time interaction for IL-10 (p = .016), associated with the increase observed for the EX+DM group at 6-weeks (88%) and 12-weeks (46%). This difference did not extend to novel analysis in the form of CRP, IL-1β, and IL-1ra.

Acute and chronic bouts of strenuous exercise is known to induce a circulatory endotoxemia, characterized by substantial increases gram-negative bacterial endotoxin (e.g., LPS), with evidence of reduced anti-endotoxin antibodies (e.g., IgM) [13, 22–24, 28]. Mechanistic explanation includes splanchnic hypoperfusion associated intestinal epithelial injury creating barrier rupture and epithelial hyperpermeability, linked with exercise-induced gastrointestinal syndrome [19]. An identified limitation in LAL chromogenic endpoint assay have given rise to surrogate markers (e.g., sCD14 and LBP) to detect the broader magnitude of luminal originated endotoxin translocation into circulation with systemic inflammatory stimulating effects [15, 17, 19]. In this study there were no significant differences in circulatory markers of bacterial endotoxin translocation following 12-weeks of progressive resistance training in any of the groups. The aforementioned studies described substantial endotoxemia as a result of acute or chronic exercise are primarily focused on endurance-based exercise [18, 19]. As there are currently a limited number of studies that determine whether resistance exercise can induce a systemic endotoxemia with or without a rise in cytokine responses to the same extent, the current study sought to investigate these effects. One study in recreationally trained young (21 ± 1 yrs) males reported and 35% increase in plasma intestinal fatty-acid binding protein (I-FABP), a, indirect marker indicative of intestinal epithelial cell injury, following 30 min of resistance training [29]. This was followed by a reduction in post-exercise protein absorption measured by a continuous intravenous infusion with l-[ring-(2)H5] phenylalanine [29]. This study suggests that resistance training can cause intestinal epithelial injury, leading to impairments in protein absorption in the post-recovery period. However, the study did not measure the gastrointestinal-systemic effects of the resistance exercise bout. Taken together, to date, the data suggests long-term chronic resistance exercise training does not induce substantial perturbations to intestinal integrity that warrant impact on systemic inflammatory responses.

Aging is associated with an increase in low-grade systemic inflammatory response, which can affect muscle metabolism and ultimately function [6, 7]. IL-10 has a reported anti-inflammatory effect and appears to play a central role in suppressing pro-inflammatory response in various tissues, including skeletal muscle [30]. We previously reported and discussed the significant increase of plasma IL-10 concentrations observed in the EX+DM group [5]. Previous studies that involve resistance training support the study’s findings of increases in plasma IL-10 following resistance training exercise [31, 32]. Considering there were no significant changes in markers suggesting compromise to the intestinal epithelial lining and associated luminal translocation of pathogenic components that may promote alterations in systemic cytokine responses (i.e., sCD14 and LBP), the current data suggests that the increase in plasma IL-10 concentration, previously reported, is likely due to a muscular cellular response. Studies *in-vivo* have indicated that IL-10 plays an integral role activating macrophages from the M1 (pro-inflammatory) to the M2 (anti-inflammatory) phenotype [32], thus supporting the observations of the current study. Previous research in skeletal muscle culture models following the activation of M2 macrophages have reported an increase in the proliferation of myoblasts and promote the proliferative stage of myogenesis [32], supporting the crucial role that IL-10 plays in the growth and development of skeletal [32]. This may explain, in part the significant increases in skeletal muscle strength previously observed in the EX+DM group compared to all other groups following 12-weeks of progressive resistance training [5]. Nonetheless, this supports that notion that regular resistance training and adequate protein intake (e.g., >1.6 gBM/day) may be needed to mitigate age-related inflammaging and sarcopenia in active older adults.

## Conclusions

In conclusion, the current study found no significant difference between any of the groups for resting intestinal epithelial integrity status markers, namely biomarkers for the identification of luminal translocations of bacterial endotoxin. Furthermore, there were no significant differences in any of the additional inflammatory cytokines measured for this study (e.g., CRP, IL-1β, and IL-1ra). The EX+DM group showed a significant anti-inflammatory response compared to EX or DM alone. The observed increase in the IL-10, reported in the previous data set, is likely due to a muscular cellular response and not an indication of exercise-associated perturbation to intestinal epithelial integrity. This suggests that active older adults that regularly engage in progressive resistance training and consume adequate protein mitigate the effects of low-grade inflammation associated with aging and may attenuate the progress of sarcopenia.

## Supporting information

S1 Data (XLSX)

S1 Table Absolute selected systemic endotoxin and inflammatory cytokine indices in response to 12-weeks progressive resistance training, with or without high-protein dairy beverage intervention. Mean and 95% CI (n = 32). Cohen’s *d*: >0.20 small effect size, *d* >0.50 moderate effect size, and *d* >0.80* large effect size.
(DOCX)

S2 Table Correlations between absolute plasma LBP, SCD14 with systemic inflammatory cytokine markers at baseline, 6-weeks, and 12-weeks. Correlation analysis were examined using Spearman rank-order correlation coefficient (r_s_) as a result of the raw data distribution (n = 32). * p< 0.05.
(DOCX)

S3 Table Correlations between relative change in plasma LBP, SCD14 with systemic inflammatory cytokine markers at baseline, 6-weeks, and 12-weeks. Correlation analysis were examined using Spearman rank-order correlation coefficient (r_s_) as a result of the raw data distribution (n = 32). * p< 0.05.
(DOCX)

S4 Table Values at baseline, week 6 and 12 total % of total energy of carbohydrate and fibre intake according to randomized allocation. (DOCX)
