# Supplementary material for: Does intestinal epithelial integrity status in response to high-protein dairy milk beverage with or without progressive resistance training impact systemic inflammatory responses in an active aging population?
Source: PLoS One. 2022 Sep 2;17(9):e0274210. doi: 10.1371/journal.pone.0274210 (PMC9439207; doi:10.1371/journal.pone.0274210)
Supplement: S1 Table — Mean and 95% CI (n = 32). Cohen’s d: >0.20 small effect size, d >0.50 moderate effect size, and d >0.80* large effect size. (DOCX) [file pone.0274210.s002.docx]

**Supplementary Table 1.** Absolute selected systemic endotoxin and inflammatory cytokine indices in response to 12-weeks progressive resistance training, with or without high-protein dairy beverage intervention.

|  | **Week 0** | **Week 6** | **Week 12** | ***d***  **0 vs 6** | ***d***  **0 vs 12** |
| --- | --- | --- | --- | --- | --- |
|  |  |  |  |  |  |
| **LBP (µg/ml)** |  |  |  |  |  |
| **EX** | 7.4 (2.6 to 12.3) | 8.3 (4.3 to 12.2) | 8.0 (4.4 to 11.6) | 0.10 | 0.07 |
| **DM** | 3.6 (1.1 to 6.2) | 4.7 (1.5 to 8.0) | 5.7 (1.6 to 9.7) | 0.23 | 0.39 |
| **EX+DM** | 8.7 (2.8 to 14.6) | 12.2 (2.5 to 22.0) | 8.6 (0.7 to 16.4) | 0.27 | 0.01 |
| **CON** | 3.5 (0.8 to 6.2) | 4.4 (0.8 to 7.9) | 3.2 (0.6 to 5.8) | 0.15 | 0.07 |
|  |  |  |  |  |  |
| **sCD14 (µg/ml)** |  |  |  |  |  |
| **EX** | 1.5 (1.1 to 1.8) | 2.0 (1.6 to 2.3) | 2.0 (1.4 to 2.5) | 0.76 | 0.66 |
| **DM** | 2.3 (1.7 to 2.9) | 2.4 (2.0 to 2.8) | 2.4 (1.9 to 2.8) | 0.10 | 0.08 |
| **EX+DM** | 2.3 (1.9 to 2.6) | 2.2 (1.8 to 2.5) | 1.9 (0.9 to 3.0) | 0.10 | 0.27 |
| **CON** | 1.4 (0.7 to 2.1) | 2.0 (1.6 to 2.3) | 1.6 (1.2 to 1.9) | 0.48 | 0.15 |
|  |  |  |  |  |  |
| **CRP (µg/ml)** |  |  |  |  |  |
| **EX** | 0.82 (0.3 to 1.4) | 1.6 (-0.3 to 3.4) | 2.3 (-1.0 to 5.5) | 0.36 | 0.41 |
| **DM** | 1.1 (.5 to 1.7) | 0.8 (0.4 to 1.1) | 1.7 (-0.2 to 3.6) | 0.38 | 0.32 |
| **EX+DM** | 1.2 (0.6 to 1.8) | 2.5 (-1.0 to 6.0) | 0.9 (0.6 to 1.1) | 0.34 | 0.36 |
| **CON** | 0.9 (0.8 to 1.0) | 0.8 (0.7 to 1.0) | 0.6 (0.4 to 0.9) | 0.26 | **0.83*** |
|  |  |  |  |  |  |
| **IL-1β (pg/ml)** |  |  |  |  |  |
| **EX** | 0.4 (-0.2 to 1.0) | 0.4 (-0.1 to 0.8) | 0.2 (-0.1 to 0.5) | 0.06 | 0.27 |
| **DM** | 0.1 (0.0 to 0.1) | 0.0 (0.0 to 0.1) | 0.4 (-0.2 to 1.0) | 0.22 | 0.55 |
| **EX+DM** | 1.0 (-0.9 to 2.8) | 1.5 (-1.1 to 4.1) | 1.3 (-1.0 to 3.7) | 0.14 | 0.10 |
| **CON** | 1.3 (-0.8 to 3.4) | 0.2 (-0.1 to 0.4) | 0.1 (-0.1 to 0.3) | 0.34 | 0.36 |
|  |  |  |  |  |  |
| **IL-1ra (pg/ml)** |  |  |  |  |  |
| **EX** | 0.6 (-0.6 t0 1.8) | 0.0 (0.0 to 0.0) | 3.0 (-2.7 to 8.6) | 0.35 | 0.39 |
| **DM** | 0.7 (-0.6 to 2.0) | 0.2 (-0.1 to 0.4) | 2.4 (-2.2 to 7.0) | 0.30 | 0.36 |
| **EX+DM** | 0.9 (-0.8 to 2.6) | 10.1 (-3.1 to 23.4) | 13.5 (-7.3 to 34.3) | 0.67 | 0.59 |
| **CON** | 6.7 (-5.5 to 18.9) | 0.5 (-0.4 to 1.5) | 0.0 (0.0 to 0.1) | 0.33 | 0.36 |
|  |  |  |  |  |  |

Mean and 95% CI (n= 32). Cohen’s *d*: >0.20 small effect size, *d* >0.50 moderate effect size, and *d* >0.80* large effect size.
