# Supplementary material for: Does intestinal epithelial integrity status in response to high-protein dairy milk beverage with or without progressive resistance training impact systemic inflammatory responses in an active aging population?
Source: PLoS One. 2022 Sep 2;17(9):e0274210. doi: 10.1371/journal.pone.0274210 (PMC9439207; doi:10.1371/journal.pone.0274210)
Supplement: S2 Table — Correlation analysis were examined using Spearman rank-order correlation coefficient (rs) as a result of the raw data distribution (n = 32). * p< 0.05. (DOCX) [file pone.0274210.s003.docx]

| **Supplementary Table 2**. Correlations between absolute plasma LBP, SCD14 with systemic inflammatory cytokine markers at baseline, 6-weeks, and 12-weeks. | | |
| --- | --- | --- |
| **Baseline** | | |
|  | LBP | sCD14 |
| CRP | r_s_= -0.092, p= 0.615 | r_s_= -0.182, p= 0.318 |
| IL-1β | r_s_= -0.013, p= 0.942 | r_s_= 0.043, p= 0.817 |
| IL-1ra | r_s_= -0.233, p= 0.200 | r_s_= -0.292, p= 0.105 |
| **6-weeks** | | |
| CRP | r_s_= -0.096, p= 0.602 | r_s_= 0.043, p= 0.816 |
| IL-1β | r_s_= -0.035, p= 0.851 | r_s_= -0.007, p= 0.971 |
| IL-1ra | r_s_= -0.338, p= 0.059 | **r_s_= -0.380, p= 0.032*** |
| **12-weeks** | | |
| CRP | r_s_= -0.054, p= 0.770 | r_s_= 0.265, p= 0.142 |
| IL-1β | r_s_= -0.124, p= 0.499 | r_s_= 0.027, p= 0.881 |
| IL-1ra | r_s_= 0.051, p= 0.773 | r_s_= -0.128, p= 0.484 |
| Correlation analysis were examined using Spearman rank-order correlation coefficient (r_s_) as a result of the raw data distribution (n= 32). * p< 0.05. | | |
