# Supplementary material for: Does intestinal epithelial integrity status in response to high-protein dairy milk beverage with or without progressive resistance training impact systemic inflammatory responses in an active aging population?
Source: PLoS One. 2022 Sep 2;17(9):e0274210. doi: 10.1371/journal.pone.0274210 (PMC9439207; doi:10.1371/journal.pone.0274210)
Supplement: S3 Table — Correlation analysis were examined using Spearman rank-order correlation coefficient (rs) as a result of the raw data distribution (n = 32). * p< 0.05. (DOCX) [file pone.0274210.s004.docx]

| **Supplementary Table 3**. Correlations between relative change in plasma LBP, SCD14 with systemic inflammatory cytokine markers at baseline, 6-weeks, and 12-weeks. | | |
| --- | --- | --- |
| **Baseline vs 6-weeks** | | |
|  | LBP | sCD14 |
| CRP | r_s_= 0.047, p= 0.799 | r_s_= -0.119, p= 0.517 |
| IL-1β | r_s_= 0.050, p= 0.787 | r_s_= 0.013, p= 0.945 |
| IL-1ra | r_s_= 0.027, p= 0.882 | r_s_= 0.033, p= 0.856 |
| **Baseline vs 12-weeks** | | |
| CRP | r_s_= -0.041, p= 0.824 | r_s_= -0.016, p= 0.930 |
| IL-1β | r_s_= 0.226, p= 0.213 | r_s_= -0.067, p= 0.714 |
| IL-1ra | r_s_= -0.035, p= 0.849 | r_s_= 0.111, p= 0.546 |
| **6-weeks vs 12-weeks** | | |
| CRP | r_s_= 0.087, p= 0.635 | r_s_= 0.072, p= 0.697 |
| IL-1β | r_s_= 0.258, p= 0.154 | **r_s_= 0.464, p= 0.007*** |
| IL-1ra | r_s_= 0.198, p= 0.278 | r_s_= -0.117, p= 0.524 |
| Correlation analysis were examined using Spearman rank-order correlation coefficient (r_s_) as a result of the raw data distribution (n= 32). * p< 0.05. | | |
