# Supplementary material for: Does intestinal epithelial integrity status in response to high-protein dairy milk beverage with or without progressive resistance training impact systemic inflammatory responses in an active aging population?
Source: PLoS One. 2022 Sep 2;17(9):e0274210. doi: 10.1371/journal.pone.0274210 (PMC9439207; doi:10.1371/journal.pone.0274210)
Supplement: S4 Table — (DOCX) [file pone.0274210.s005.docx]

| **Supplementary Table 4.** Values at baseline, week 6 and 12 total % of total energy of carbohydrate and fibre intake according to randomized allocation. | | | | |
| --- | --- | --- | --- | --- |
|  | **DM**  **n=7** | **EX+DM**  **n=8** | **EX**  **n=8** | **CON**  **n=9** |
| **Dietary Carbohydrates %TE** | | | | |
| Baseline | 40.0 (35.0-45.9) | 35.3 (25.0-47.3) | 39.0 (31.2-50.0) | 35.0 (10.5-45.2)^**^ |
| 6 weeks | 54.0 (44.0-72.3)^**^ | 50.0 (45.5-54.4)^**b^ | 41.0 (34.0-49.0)^**a^ | 36.5 (10.4-59.3)^**^ |
| 12 weeks | 52.3 (47.0-56.0)^**^ | 49.4 (45.5 -54.3)^**b^ | 41.0 (29.0-50.0)^**a^ | 37.3 (14.5-71.1)^**^ |
| **Fibre, g/day** | | | | |
| baseline | 28.1 (15.0-37.2) | 21.4 (9.1-48.2) | 29.0 (16.6-65.6) | 33.1 (17.0-52.0) |
| 6 weeks | 33.0 (21.2-46.0) ^**^ | 28.1 (22.0-37) ^**^ | 33.0 (13.3-72.2) ^**^ | 33.2 (8.6-55.0) |
| 12 weeks | 35.0 (27.0-43.3) ^**^ | 28.5 (23.0-33.4) ^**^ | 31.0 (18.0-37.2) ^**^ | 30.2 (12.3-48.5) ^**^ |
| Values are shown in the mean (95% CI)  Abbreviations: RE: total energy. Within group changes ** p<0.01 and p<0.05 vs. baseline; between-group changes ^a^P<0.01 vs DM, ^b^P<0.01 vs EX | | | | |
